# Mutation analysis of the *SLC4A11* gene in Indian families with congenital hereditary endothelial dystrophy 2 and a review of the literature

**Published:** 2013-08-02

**Authors:** Srinivas Gopinath Kodaganur, Saketh Kapoor, Avinash M. Veerappa, Sagar Jagannath Tontanahal, Astha Sarda, S. Yathish, D. Ravi Prakash, Arun Kumar

**Affiliations:** 1 Minto Eye Hospital, Bangalore Medical College and Research Institute, Bangalore India; 2 Department of Molecular Reproduction, Development and Genetics, Indian Institute of Science, Bangalore, India

## Abstract

**Purpose:**

Congenital hereditary endothelial dystrophy 2 (CHED2) is an autosomal recessive disorder caused by mutations in the solute carrier family 4, sodium borate transporter, member 11 (*SLC4A11*) gene. The purpose of this study was to identify the genetic cause of CHED2 in six Indian families and catalog all known mutations in the *SLC4A11* gene.

**Methods:**

Peripheral blood samples were collected from individuals of the families with CHED2 and used in genomic DNA isolation. PCR primers were used to amplify the entire coding region including intron-exon junctions of *SLC4A11*. Amplicons were subsequently sequenced to identify the mutations.

**Results:**

DNA sequence analysis of the six families identified four novel (viz., p.Thr262Ile, p.Gly417Arg, p.Cys611Arg, and p.His724Asp) mutations and one known p.Arg869His homozygous mutation in the *SLC4A11* gene. The mutation p.Gly417Arg was identified in two families.

**Conclusions:**

This study increases the mutation spectrum of the *SLC4A11* gene. A review of the literature showed that the total number of mutations in the *SLC4A11* gene described to date is 78. Most of the mutations are missense, followed by insertions-deletions. The present study will be helpful in genetic diagnosis of the families reported here.

## Introduction

Congenital hereditary endothelial dystrophy (CHED) is a rare inherited disorder of the corneal endothelium, characterized by corneal opacification and nystagmus. CHED is usually evident at the time of birth or in the early years of life. This disorder is due to the malfunction and degeneration of the corneal endothelium that lead to corneal edema, especially of the stroma, and give the cornea the appearance of ground glass [1]. The condition is known to occur in two genetic forms: autosomal dominant (CHED1, MIM# 121700) and autosomal recessive (CHED2, MIM# 217700), the latter more severe and usually more common. CHED1 and CHED2 have been mapped to chromosome 20 at two distinct loci [2,3]. Vithana et al. [4] identified the CHED2 gene solute carrier family 4, sodium borate transporter, member 11 (*SLC4A11*) through a positional candidate gene approach. SLC4A11, also known as bicarbonate transporter-related protein-1 (BTR1), is a member of the SLC4 family of bicarbonate transporters. In humans, the SLC4A and SLC26 families are the main bicarbonate transporters [5]. The *SLC4A11* gene consists of 18 coding exons and is expressed in several organs and tissues, including the eye, blood, lung, ovary, colon, mouth, embryonic tissue, pancreas, kidney, skin, cranial nerve, ascites, prostate, and brain. The gene encodes an 891-amino-acid-long protein with a calculated molecular mass of 100 kDa, which contains 14 transmembrane domains along with multiple intracellular phosphorylation sites and two extra cellular N-glycosylation sites [6]. Homozygous mutations in *SLC4A11* have also been shown to cause another rare autosomal recessive disorder, corneal dystrophy and perceptive deafness (CDPD, MIM# 217400) or Harboyan syndrome [7]. Interestingly, heterozygous mutations in *SLC4A11* cause Fuchs endothelial corneal dystrophy-4 (FECD4, MIM# 613268) [8-10].

Mutations in *SLC4A11* have been reported in families with CHED2 from different populations, including India [4,11-19]. We reported earlier two homozygous mutations in the *SLC4A11* gene in two families with CHED2 ascertained at the Minto Eye Hospital, Bangalore. Here, we report on the mutation analysis of this gene in six additional families with CHED2 identified at the same hospital. We have also reviewed the literature on the contribution of this gene in CHED2, FECD4, and CDPD.

## Methods

### Familie**s**

**Figure 1:**
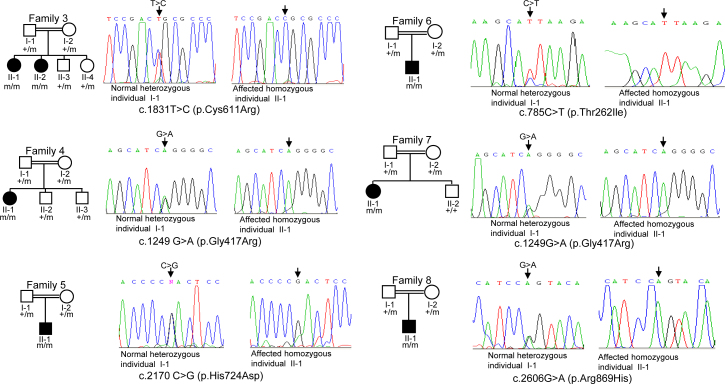
Deoxyribonucleic acid sequence analysis of individuals. Sequencing chromatograms of the heterozygous parents and affected homozygous individuals from family 3, 4, 5, 6, 7, and 8 are shown. Arrows mark the nucleotide change in a heterozygous state in parents and in a homozygous state in affected individuals. + and m denote the wild type and the mutant alleles.

**Figure 2:**
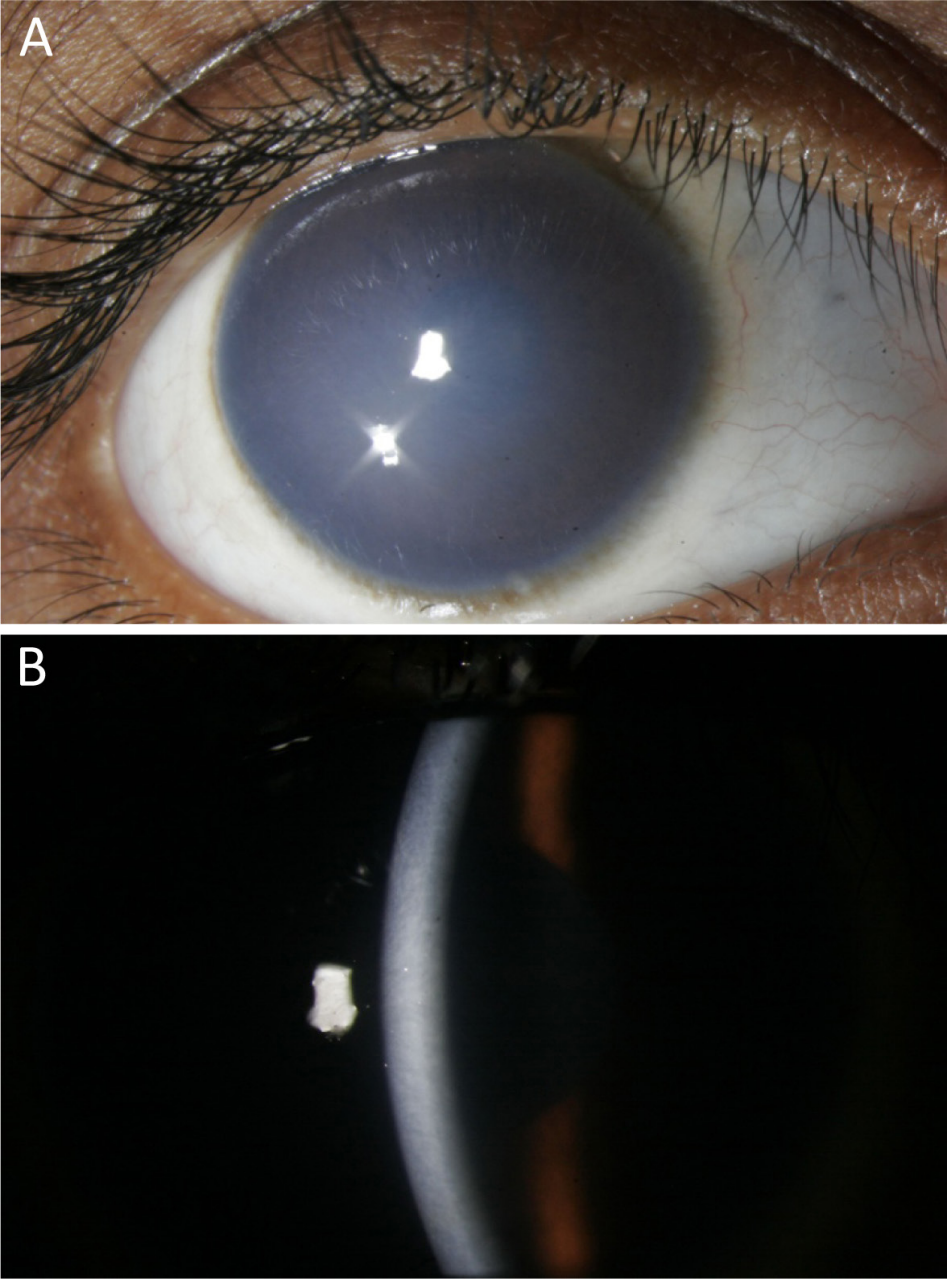
Clinical features of affected individual II-1 from family 7. **A**: Cornea showing opacification. **B**: Slit-lamp examination of the cornea showing thickening and opacification.

We recruited seven patients from six consanguineous families (Figure 1), three boys and four girls, aged 3-6 years, at the Minto Eye Hospital, Bangalore, Karnataka. Consanguinity was due to maternal uncle and niece marriage in all the families. All family members were examined in detail by D. Ravi Prakash and S. Yathish. Their state of health at the time of recruitment was good with the exception that all affected individuals from six families had congenital bilateral cloudy cornea (Figure 2). None of the parents had cloudy cornea or any other systemic involvement.

### Genetic analysis

For genetic analysis, 3–5 ml of peripheral blood sample was drawn from each individual in a Vacutainer EDTA tube (Becton Dickinson, Franklin Lakes, NJ) and used for genomic DNA isolation using a Wizard genomic DNA extraction kit (Promega, Madison, WI). This research followed the tenets of the Declaration of Helsinki and the guidelines of the Indian Council of Medical Research, New Delhi. To determine if CHED2 in these families is due to mutations in the *SLC4A11* gene, the entire coding region of the *SLC4A11* gene was amplified using primers that amplify all coding exons and their intron-exon junctions [12]. Mutations were identified by sequencing the PCR products from one affected individual from each family on an ABIprism A310-automated sequencer (Life Technologies, Carlsbad, CA). PCR was performed in a total volume of 25 µl containing 50 ng of genomic DNA, 1.5 mM MgCl_2_, 200 µM of each deoxynucleotide triphosphate, 1X buffer, and 1 unit of *Taq* DNA polymerase (Sigma-Aldrich, Bangalore, India) using a PTC-100 thermocycler (MJ Research, Waltham, MA). Before sequencing, the PCR products were purified using a GenElute gel extraction kit (Sigma-Aldrich, St. Louis, MO). Once a mutation was identified, all members of the family were sequenced to identify the mutation. Allele-specific PCR was performed to determine if a specific mutation was present in 50 ethnically matched normal controls (Table 1).

**Table 1:** Primers used for mutation analysis in normal controls by allele-specific PCR.

**Mutation**	**Primer sequence (5′-3′)**	**Tm (°C)**	**Amplicon size (bp)**
c.1831T>C	F:GCGTGCGAGAGATCCTGTCCGACC 14R*: AGTAGGGGACAGGCTACTGCTATGCC	70	168
c.1249G>A	F:GACCATAGCCGGGCAGAGCATCA 11R*:GGGCTGAACCAGATCCCAAGCCTTGA	66	379
c.2170C>G	F:CTTGGATCCATGCCGCCTACCCCGA 16R*:GGCCAGAGGCTCCCCACTCCTCAG	61	149
c.785C>T	8F*:CCCGGGCAGGGCCTCCTCTGTTTC R:GCGCGCCACCTCCATCGCAGTCTTAA	72	86

Abbreviations: F, forward primer; R, reverse primer, Tm, annealing temperature; and, bp, base pairs. *Primers are described in Kumar et al. [12].

To find the functional significance of the mutated amino acid residues, SLC4A11 protein sequences from different species were aligned by the ClustalW2 program. To predict the effect of mutations on SLC4A11 function, we used two bioinformatics programs: PolyPhen-2 and MutationTaster. The output score from the PolyPhen-2 program ranges from 0 to a positive number, where 0 is neutral, and a high positive number is damaging to protein function. The output from the MutationTaster program is a p (probability) value. A p value close to 1 indicates the high “security” of the prediction that the mutation is damaging to protein function.

## Results and Discussion

DNA sequence analysis of the *SLC4A11* gene showed four novel mutations, c.1831T>C (p.Cys611Arg), c.1249G>A (p.Gly417Arg), c.2170C>G (p.His724Asp), and c.785C>T (p.Thr262Ile) in families 3, 4, 5, and 6, respectively, and a known mutation, c.2606G>A (p.Arg869His), in family 8 in a homozygous state (Figure 1; Table 2). Interestingly, the c.1249G>A (p.Gly417Arg) mutation was also observed in family 7 (Figure 1).

**Table 2:** Effect of novel mutations on SLC4A11 function by the *in silico* analysis.

Sl.#	Family	Mutation	PolyPhen-2 score	Mutation Taster score
1	Family 3	c.1831T>C	Probably damaging with	Disease causing with
		(p.Cys611Arg)	a score of 0.99	a p value of 0.99
2	Family 4 and	c.1249 G>A	Probably damaging with	Disease causing with
	Family 7	(p.Gly417Arg)	a score of 1	a p value of 0.99
3	Family 5	c.2170 C>G	Probably damaging with	Disease causing with
		(p.His724Asp)	a score of 1	a p value of 0.99
4	Family 6	c.785C>T	Probably damaging	Disease causing with
		(p.Thr262Ile)	with a score of 1	a p value of 0.99

**Figure 3:**
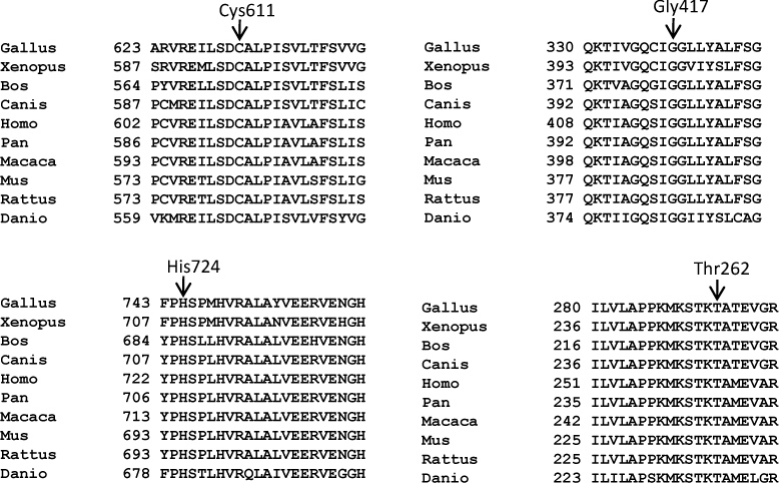
Conservation of the amino acid residues across different species. Arrows mark the conservation of mutated amino acid residues Cys611, Gly417, His724, and Thr262 across different species in SLC4A11. The number refers to the position of the amino acid residue.

Based on the following criteria, we considered the four novel changes mutations. 1) The changes were segregating in the family (Figure 1). 2) The changed amino acids were highly conserved across species (Figure 3). 3) The changes were not observed in 50 normal controls (data not shown). 4) The PolyPhen-2 program predicted all four changes would probably be damaging (Table 2). 5) The MutationTaster program predicted the four changes would be disease causing (Table 2).

We performed a literature review to catalog all the mutations described to date in the *SLC4A11* gene. With the four novel mutations described in the present study, the total number of mutations in this gene reaches 78 (Table 3). These include 42 missense, nine nonsense, four splice site, and 23 insertion-deletion mutations (Table 3). The mutations are scattered across the gene (Table 3), suggesting that its entire coding region needs to be sequenced in an affected individual to identify the mutation.

**Table 3:** Known mutations in the *SLC4A11* gene.

**Sl.** **no.**	**Mutation**	**Exon/ intron (IVS)**	**Nature of mutation**	**State of zygosity**	**Effect on protein**	**Phenotype**	**Number and ethnic origin of family**	**Reference**
1	c.99_100delTC (p.S33SfsX18)	2	Deletion	Heterozygous	Truncation of protein and addition of novel amino acids	FECD4	1 Chinese	[8]
2	c.140delA(p.Y47SfsX69)	2	Deletion	Homozygous	Truncation of protein and addition of novel amino acids	CHED2	1 Indian	[11]
3	c.246_247delTTinsA (p.R82RfsX33)	2	Indel	Homozygous	Truncation of protein and addition of novel amino acids	CHED2	1 Indian	[14]
4	c.306delC (p.G103VfsX13)	3	Deletion	Compound heterozygous with an unknown second mutation	Truncation of protein and addition of novel amino acids	CHED2	1 Indian	[11]
5	c.334C>T (p.R112X)	3	Nonsense	Homozygous and compound heterozygous with c.2318C>T (p.773L) and c.1751C>A (p.T773K)	Truncation of protein	CHED2	3 Indian	[11]
6	c.353_356delAGAA (p.K118TfsX11)	4	Deletion	Homozygous	Truncation of protein and addition of novel amino acids	CHED2	2 Indian	[4]
7	c.374G>A (p.R125H)	4	Missense	Homozygous	May have an effect on N-terminal cytoplasmic domain	CHED2	1 Indian	[16]
8	c.427G>A (p.E143K)	4	Missense	Homozygous	May have an effect on N-terminal cytoplasmic domain	CHED2	1 Indian	[13]
9	c.520delGCTTCGCC (p.R158fs)	4	Out-of-frame deletion	Homozygous	Truncation of protein	CHED2	1 Saudi Arabian	[18]
10	c.473_481delGCTTCGCCAinsC (p.R158PfsX3)	4	Indel	Homozygous	Truncation of protein and addition of novel amino acids, absence of all TMD	CHED2	1 Indian	[16]
11	c.473_480del8 bp (p.R158QfsX4)	4	Deletion	Homozygous	Truncation of protein and addition of novel amino acids	CHED2 and CDPD	2 Indian, 1 Gipsy (Eastern European)	[7,11]
12	c.478G> A (p.A160T)	4	Missense	Homozygous	May have an effect on N-terminal cytoplasmic domain	CHED2	2 Indian	[14,16]
13	c.501G>C (p.E167D)	4	Missense	Heterozygous	Reduction in the mature 120 kDa form, with addition of 100 kDa species	FECD4	Northern European (No. of families not mentioned)	[10]
14	c.618_619delAG (p.V208AfsX38)	5	Deletion	Homozygous	Truncation of protein and addition of novel amino acids	CHED2	2 Indian	[11]
15	c.625C>T (p.R209W)	5	Missense	Homozygous	May have an effect on N-terminal cytoplasmic domain	CHED2	2 Indian	[11]
16	c.637T>C (p.S213P)	5	Missense	Compound heterozygous with c.2566A>G (p.M856V)	May have an effect on N-terminal cytoplasmic domain	CDPD	1 Sephardi Jewish	[7]
17	c.638C>T (p.S213L)	5	Missense	Homozygous	May have an effect on N-terminal cytoplasmic domain	CHED2	1 Indian	[11]
18	c.654 (−97)_c.778 (−1488)del698 (p.C218KfsX49)	5–6	Deletion	Homozygous	Truncation of protein and addition of novel amino acids, absence of all TMDs	CHED2	1 Indian	[16]
19	c.743G>A (p.S232N)	6	Missense	Compound heterozygous with c.1033A>T (p.Arg329X)	Loss of function or membrane localization	CHED2	1 US family of Chinese ancestry	[15]
20	c.697C>T (p.R233C)	6	Missense	Homozygous	May have an effect on N-terminal cytoplasmic domain	CHED2	1 Indian	[11]
21	c.720G>A (p.W240X)	6	Nonsense	Homozygous	Truncation of protein	CHED2	1 British	[13]
22	c.785C>T (p.T262I)	6	Missense	Homozygous	Damaging to protein function	CHED 2	1 Indian	Present study
23	c.806C>T (p.A269V)	7	Missense	Homozygous	May have an effect on N-terminal cytoplasmic domain	CHED2	2 Indian	[16]
24	c.812C>T (p.T271M)	7	Missense	Homozygous	May have an effect on N-terminal cytoplasmic domain	CHED2	1 Saudi Arabian	[17]
25	c.845G>C (p.R282P)	7	Missense	Heterozygous	Immature protein	FECD4	Northern European (No. of families not mentioned)	[10]
26	c.859_862delGAGA insCCT (p.E287PfsX21)	7	Indel	Homozygous	Truncation of protein and addition of novel amino acids, absence of all TMDs	CHED2	1 Indian	[12]
27	c.878_889del12 p.E293_E296del	7	Deletion	Homozygous	May have an effect on N-terminal cytoplasmic domain	CHED2	1 Indian	[11]
28	c.1033A>T (p.R329X)	7	Nonsense	Compound heterozygous with c.743G>A (p.Ser232Asn)	Premature truncation of the transcript	CHED2	1 US family of Chinese ancestry	[15]
29	c.996+26C_+44Cdel19	IVS-7	Deletion	Homozygous	Not Known	CHED2	2 Indian	[11]
30	c.1044+25del19nt	IVS-7	Deletion	Homozygous	Not known	CHED2	1 Saudi Arabian	[18]
31	c.1091–1G>C	IVS-8	Splice site	Homozygous	Not known	CHED2	1 Indian	[11]
32	c.1156T>C (p.C386R)	9	Missense	Homozygous	Disruption of TMD 1	CHED2	4 Indian	[13,16,19]
33	c.1228G>C (p.G394R)	9	Missense	Homozygous	Disruption of TMD1	CHED2	1 Saudi Arabian	[18]
34	c.1195G>A (p.E399K)	9	Missense	Heterozygous	Aberrant glycosylation and cellular localization	FECD4	1 Indian	[8]
35	c.1202C>A (p.T401L)	9	Missense	Compound heterozygous with c.1418T>G (p.L473R)	Not known	CHED2	1 Indian	[11]
36	c.1249 G>A (p.G417R)	10	Missense	Homozygous	Damaging to protein function	CHED2	2 Indian	Present study
37	c.1253G>A (p.G418D)	10	Missense	Homozygous	Disruption of TMD 2	CHED2	1 Indian, 1 Saudi Arabian	[11,18]
38	c.1317_1322del6ins8 (p.L440VfsX6)	10	Indel	Homozygous	Truncation of protein and addition of novel amino acids	CHED2	1 Indian	[11]
39	c.1378_1381delTACGinsA (p.Y460_A461 delinsT)	11	Indel	Homozygous	Not known	CDPD	1 Dominican Republican	[7]
40	c.1391G>A (p.G464D)	11	Missense	Homozygous	Conformation change	CHED2	3 Pakistani	[4]
41	c.1463G>A (p.R488K)	11	Missense	Homozygous	Not known	CDPD	1 Moroccan	[7]
42	c.1466C>T (p.S489L)	12	Missense	Homozygous	Conformation change	CHED2	1 Pakistani, 1 Indian	[4,11]
43	c.1577A>G (p.Y526C)	12	Missense	Heterozygous	Partial loss of localization at the membrane	FECD4	Northern European (No. of families not mentioned)	[10]
44	c.1704_1705delCT (p.H568HfsX177)	13	Deletion	Homozygous	Truncation of protein and addition of novel amino acids	CHED2	1 Indian	[14]
45	c.1723G>A (p.V575M)	13	Missense	Heterozygous	Partial loss of localization at the membrane	FECD4	Northern European (No. of families not mentioned)	[10]
46	c.1748G>A (p.G583D)	13	Missense	Heterozygous	Immature protein	FECD4	Northern European (No. of families not mentioned)	[10]
47	c.1751C>A (p.T584K)	13	Missense	Homozygous and compound heterozygous with c.334C>T (p.Arg112X)	Disruption of TMD 6	CHED2	2 Indian	[11]
48	c.1813C>T (p.R605X)	14	Nonsense	Homozygous and compound heterozygous with an unknown second mutation	Truncation of protein	CHED2	6 Indian	[4,11,14]
49	c.1831T>C (p.C611R)	14	Missense	Homozygous	Damaging to protein function	CHED2	1 Indian	Present study
50	c.1894G>T (p.E632X)	14	Nonsense	Homozygous	Truncation of protein	CHED2	2 Indian	[11,14]
51	IVS15 −6 _ −16 delins GGCCGGCCGG	IVS-15	Indel	Homozygous	Inactivation of splice acceptor site	CHED2	1 Indian	[4]
52	c.2014_2016delTTC (p.F672del)	15	In-frame deletion	Homozygous	Disruption of TMD8	CHED2	1 Indian	[12]
53	c.2067–6_-16delinsGGCCGGCCGG	IVS-15	Splice site	Homozygous	Inactivation of an acceptor splice site	CHED2	1 Indian	Cited in [16]
54	c.2114+1G>A	IVS-15	Donor Splice site	Homozygous	Inclusion of intron 15	CHED2	1 Saudi Arabian	[18]
55	c.2126G>A (p.G709E)	15	Missense	Heterozygous	Aberrant glycosylation and cellular localization	FECD	1 Chinese	[8]
56	c.2170 C>G (p.His724Asp)	15	Missense	Homozygous	Damaging to protein structure	CHED2	1 Indian	Present study
57	c.2224G>A (p.G742R)	16	Missense	Heterozygous	Reduction in the mature 120-kDa form, with addition of 100-kDa species	FECD	Northern European (No. of families not mentioned)	[10]
58	c.2233_2240dup TATGACAC (p.T747TfsX6)	16	Duplication	Compound heterozygous with c.2528T>C (p.L843P)	Aberrantly truncated protein of 916 residues	CDPD	1 South American Indian	[7]
59	c.2236C>T (p.R757X)	16	Nonsense	Homozygous	Protein truncation	CHED2	2 Saudi Arabian	[18]
60	c.2240 +1G>A	IVS-16	Splice site	Homozygous and compound heterozygous with an unknown second mutation	Inactivation of splice donor site	CHED2	1 British, 1 Indian	[13,19]
61	c.2261C>T (p.T754M)	17	Missense	Heterozygous	Aberrant glycosylation and cellular localization	FECD4	1 Chinese	[8]
62	c.2263C>T (p.R755W)	17	Missense	Homozygous	Disruption of TMD 11	CHED2	3 Indian	[11,13,16]
63	c.2264G>A (p.R755Q)	17	Missense	Homozygous and compound heterozygous with c.2623C>T (p.Arg875X)	Conformation change	CHED2	4 Indian, 1 Myanmar	[4,11,13,14]
64	c.2318C>T (p.P773L)	17	Missense	Homozygous and compound heterozygous with c.334C>T (p.R112X)	Disruption of TMD 11	CHED2	3 Indian	[11,16]
65	c.2389_2391delGAT (p.D797del)	17	Deletion	Homozygous	Disruption of TMD 12	CHED2	1 Indian	[11]
66	c.2398C>T (p.Q800X)	17	Nonsense	Compound heterozygous with c.2437–1G>A	Truncation of protein	CHED2	1 British	[13]
67	c.2407C>T (p.Gln803X)	17	Nonsense	Homozygous	Truncation of protein	CHED2	1 Indian	[11]
68	c.2411G>A (p.R804H)	18	Missense	Homozygous	Conformation change	CHED2	1 Indian family	[14]
69	c.2420delTinsGG (p.L807RfsX71)	18	Missense	Homozygous	Truncation of protein and addition of novel amino acids	CHED2	1 Indian family	[14]
70	c.2423_2454del 32nt (p.Leu808ArgfsX110)	17	Deletion	Compound heterozygous with c.2528T>C (p.Leu843Pro)	Aberrantly truncated protein of 916 residues	CDPD	1 Dutch	[7]
71	c.2470G>A (p.V824M)	18	Missense	Homozygous	Not known	CHED2	6 Indian	[7,11,19]
72	c.2498C>T (p.T833M)	18	Missense	Homozygous	Conformation change	CHED2	2 Indian	[14]
73	c.2500G>A (p.G834S)	18	Missense	Heterozygous	Immature protein	FECD	Northern European (No. of families not mentioned)	[10]
74	c.2506 C>T (p.Q836X)	18	Nonsense	Compound heterozygous with c.2318C>T (p.P773L)	Truncation of protein	CHED2	1 Indian	[16]
75	c.2518–2520 delCTG (p.L840del)	18	In-frame deletion	Homozygous	Disrupts the appropriate assembly or localization of protein in the membrane	CHED2	1 Indian	[19]
76	c.2605C>T (p.R869C)	18	Missense	Homozygous	Conformation change	CHED2	3 Indian, 1 Middle Eastern	[4,11,13]
77	c.2606G>A (p.R869H)	18	Missense	Homozygous	Damaging to protein structure	CHED2	3 Indian	[14], Present study
78	c.2618T>C (p.L873P)	19	Missense	Homozygous	Disruption of TMD 14	CHED2	1 Indian	[16]

CDPD is a degenerative corneal disorder characterized by the association of congenital hereditary endothelial dystrophy with progressive sensorineural hearing loss. The ocular manifestations in CDPD include diffuse bilateral corneal edema occurring with severe corneal clouding, blurred vision, visual loss, and nystagmus, which are usually present at birth or within the neonatal period and are indistinguishable from CHED2. The sensorineural hearing loss is slowly progressive and can be identified only during the second decade of life [20]. As stated, homozygous mutations in *SLC4A11* cause not only CHED2 but also CDPD. One of the mutations, c.473_480del8bp (p.R158QfsX4), causes CHED2 and CDPD (Table 3).

Why some individuals also develop perceptive deafness along with corneal dystrophy due to mutations in *SLC4A11* is unclear. However, it could be due to an additional environmental effect and/or genetic modifiers. Morris et al. [21] showed differential expression of *SLC4A11* in the inner ear of mice specifically in the region of the stria vascularis. Taking this fact into account, Desir et al. [7] postulated that corneal dystrophy and perceptive deafness might have a common origin in the neural crest cells from which the stria vascularis and the corneal endothelium develop. Further, four mutations (p.S213P, p.Y460_A461 delinsT, p.R488K, and p.Leu808ArgfsX110) are specific to only CDPD (Table 3), and none of the 11 heterozygous mutations causing FECD (FECD4) are found in patients with CHED2 and CDPD (Table 3). FECD is a progressive degeneration of the corneal endothelium leading to thickened Descemet’s membrane, a collagen-rich basal lamina secreted by the endothelium, and reduced vision. In patients with FECD, corneal endothelial cells die, as a result of which bumps called guttae form on the back of the cornea. This causes the cornea to swell and distort vision, resulting in pain and severe visual impairment [8,22].

Why some heterozygous mutations in *SLC4A11* cause FECD4 is also not clear. However, it could be speculated on. The involvement of *SLC4A11* in various corneal dystrophies suggests a significant genetic overlap occurs across several corneal dystrophies and they might share a common pathomechanism [10]. Moreover, the characteristic abnormal posterior non-banded zone of the Descemet’s membrane, which represents an abnormal function of the corneal endothelium in CHED2 and FECD4, underlies the importance of the SLC4A11 protein for the proper development and differentiation of the corneal endothelium and may explain how the same gene can be involved in the pathogenesis of CHED2 and FECD4 [8,22]. In addition, a combination of mechanisms may be at play, with partial loss of function and gradual accumulation of the aberrant misfolded protein having a role in FECD4 pathology [8].

It is not surprising to find mutations in *SLC4A11* causing three different disorders. Similar to *SLC4A11*, mutations in the same gene are known to cause different disorders. For example, null mutations in *CEP290* (*NPHP6*) cause Meckel syndrome (MKS4, MIM# 611134) [23], Bardet-Biedl syndrome (BBS14, MIM# 209900) [24], and Joubert syndrome (JBTS5, MIM# 610188) [25,26], while hypomorphic mutations in the same gene lead to Leber congenital amaurosis (LCA10, MIM# 611755) [27].

In summary, we have identified four novel mutations in the *SLC4A11* gene in the present study. With the four novel mutations reported here, the total number of mutations described to date in *SLC4A11* reaches 78. Further, this information will be useful for providing rapid prenatal diagnosis and genetic counseling to families and their relatives.
